# Treatment of Acetabulum Anterior Wall Fractures Using the Modified Stoppa Approach

**DOI:** 10.7759/cureus.47770

**Published:** 2023-10-26

**Authors:** İsmail G Şahin, Fatih İ Can, Emre Gültaç, Rabia M Kilinc, Nevres H Aydoğan, Cem Y Kilinc

**Affiliations:** 1 Orthopedics and Traumatology, Muğla Sıtkı Koçman Training and Research Hospital, Muğla, TUR; 2 Orthopedics and Traumatology, Muğla Sıtkı Koçman University, Muğla, TUR; 3 Radiology, Muğla Sıtkı Koçman University, Muğla, TUR

**Keywords:** trauma, technique, surgery, fractures, acetabulum

## Abstract

Introduction

The objective of this study was to describe the modified Stoppa approach for anterior wall fracture fixation and to present our radiological and functional results of this approach.

Materials and methods

Between April 2013 and December 2019, 256 acetabular fractures operated with the modified Stoppa approach in our clinic were retrospectively reviewed, and 11 patients who were operated for anterior acetabular wall fractures with at least two years of follow-up were included in the study.

Results

The median amount of bleeding during surgery was found to be 450 ml (200-800), and the median operation time was 120 minutes (90-180). The modified Merle d'Aubigné and Postel Hip Score (MDS), modified Harris Hip Score (HHS), Matta's Reduction Criteria (MRC), and Kellgren-Lawrence Classification (KLC) outcomes are similar to the anterior surgical approach.

Conclusion

We suggest that the modified Stoppa approach can be safely used in the surgical treatment of isolated anterior acetabular fractures due to its short operative duration, low amount of bleeding, low complication rate, and favorable radiological and clinical results. The modified Stoppa procedure is a considerable alternative to the ilioinguinal approach in anterior acetabular fractures.

## Introduction

Acetabular fractures usually occur due to high-energy traumas such as falling from a height, motor vehicle accidents, and crushing injuries, and their incidence is increasing day by day [1]. The management of these fractures is one of the most challenging issues of current orthopedic trauma surgery due to the complexity of the major pelvic neurovascular structures and the fracture itself, the difficulty of reduction-osteosynthesis of complex fractures, and the steep learning curve of the procedures. Surgical treatment of acetabular fractures was popularized by the classification and anterior (ilioinguinal) approach developed by Judet and Letournel in 1964, and it is increasing gradually thanks to the development of imaging methods, increasing surgical experience, and new surgical approaches [2-4].

The approach chosen in the surgical treatment of acetabular fractures should provide a wide field of view, allowing appropriate anatomical reconstruction and osteosynthesis. The modified Stoppa (anterior intrapelvic extraperitoneal) approach was initially used for the treatment of acetabular fractures involving a quadrangular surface with a more limited field of view. With the increase of surgical experience, this approach is being successfully used in the treatment of various types of fractures such as isolated anterior wall fractures, isolated anterior column fractures, anterior column-related posterior hemi transverse fractures, transverse fractures, T-type fractures, double column fractures, and selective posterior column fractures in addition to quadrangular surface fractures [5-9].

Currently, there are publications stating that the modified Stoppa approach has a better anatomical reduction chance and lower complication rates compared to the ilioinguinal approach [10-12]. In addition, acetabulum anterior wall fractures have also been reported as complications of total hip replacement surgery, and anterior wall fractures treated with a modified Stoppa approach simultaneously with total hip replacement surgery are also presented in the literature [13].

Isolated anterior acetabular fractures account for approximately 10% of all acetabular fractures, and the anterior (ilioinguinal) approach is the mainstay of treatment [10-13]. Although it is stated that modified Smith-Peterson and anterior intrapelvic (modified Stoppa) approaches can be used in the surgical treatment of these fractures, there is no detailed publication describing the surgical technique of using modified Stoppa.

The ilioinguinal approach is the gold standard surgical approach in acetabulum anterior wall fractures and is safely performed. Our aim in this study is to describe the new technique in which the modified Stoppa approach can be used safely in fractures of the anterior wall of the acetabulum and to present our surgical results using the technique we described.

## Materials and methods

**Figure 1:**
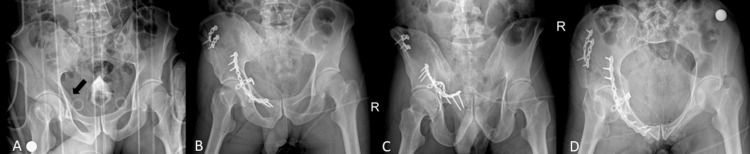
Preoperative and postoperative radiographs A) preoperative pelvis anterior-posterior (AP) radiograph, B) postoperative pelvis AP radiograph, C) postoperative pelvis outlet radiograph, D) postoperative pelvis inlet radiograph

**Figure 2:**
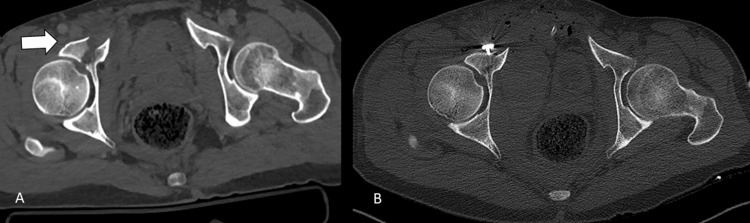
Computed tomography sections A) preoperative, B) postoperative

**Figure 3:**
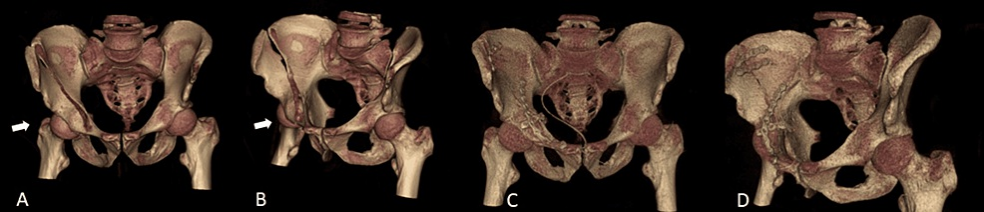
Three-dimensional reconstructions A) preoperative pelvis anterior view, B) preoperative iliac oblique view, C) postoperative pelvis anterior view, D) postoperative iliac oblique view

Acetabular fractures, which were operated using the modified Stoppa approach in our clinic between April 2013 and December 2019, were scanned retrospectively through the medical records after the approval of the ethics committee. After ethics committee approval, an archive search was conducted between January and June 2020, and only patients who underwent surgery using the modified Stoppa approach due to acetabulum anterior wall fracture were included in the study. Cases other than acetabular, anterior wall fractures, and cases followed for less than two years after surgery were excluded from the study. All cases outside the exclusion criteria were included in the study, and a sample was formed. Due to the small sample group, statistical comparisons were calculated with non-parametric analyses. As a result of the scan, it was determined that 26 (10.16%) of 256 acetabulum fractures treated surgically had isolated acetabulum anterior injury. Among these 26 patients, 15 patients (5.86%) had isolated anterior colon fracture, eight patients (3.13%) had anterior column and anterior wall fracture, and three patients had isolated anterior wall fracture (1.18%). Eleven out of 26 patients with acetabulum anterior wall fracture were included in the study (Figures 1-3)

Patient information was scanned from the printed and digital archives through the hospital records, and the demographic data of the patients, the time of the operation, the duration of the operation, and the amount of bleeding were determined (Table 1). Surgical procedures were performed by a single surgeon, the senior surgeon of the trauma team.

**Table 1:** Demographic and surgery data BMI - body mass index, FT - fracture type, AW - acetabulum anterior wall fracture, AC - acetabulum anterior colon fracture, ST - surgery time (min)

Patient	Age	Gender	Height(cm)	Weight(kg)	BMI	FT	Bleeding (ml)	ST
1	37	Male	165	70	25.71	AW+AC	500	120
2	41	Female	167	55	19.72	AW+AC	750	120
3	28	Male	175	88	28.73	AW	450	90
4	62	Male	159	65	25.71	AW+AC	300	120
5	45	Female	172	70	23.66	AW+AC	450	150
6	56	Male	162	65	24.77	AW+AC	250	105
7	19	Male	170	75	25.95	AW+AC	550	105
8	33	Male	168	65	23.03	AW	200	90
9	49	Male	165	75	27.55	AW	350	90
10	40	Male	182	105	31.70	AW+AC	800	150
11	25	Male	174	85	28.08	AW+AC	350	180

The follow-up procedure for acetabulum fractures is standardized in our clinic. Hemovac drains were removed on the second day after surgery. The amount of bleeding of the patients was recorded based on the work of Açan et al. [14]. Patients received subcutaneous low molecular weight heparin therapy for six weeks as a prophylactic agent for deep vein thrombosis. Patients received range of motion (ROM) exercises and in-bed isometric exercises after the postoperative first day. Three weeks later, mobilization was allowed by bearing weight on the contralateral side. Starting from the second month, partial weight bearing is allowed to the fractured side. After the second month, patients who had residual ROM limitations were referred to the physical therapy unit.

At the first follow-up examination at the end of the second postoperative year, clinical results were evaluated using modified Merle d'Aubigné and Postel hip score (Md'APs) [15,16] and modified Harris Hip score (HHS) [17,18], and radiological results were evaluated using Matta's reduction criteria [19] and Kellgren-Lawrence radiology criteria (K-L) [20] (Table 2).

**Table 2:** Functional and radiological results mMd'AP - modified Merle d'Aubigné and Postel hip score

Patient	Age	mMd'AP	Harris Hip score	Matta reduction	Kellgren-Lawrence	Heterotopic ossification
1	37	Excellent	Excellent	Anatomical	Grade 0	None
2	41	Fair	Fair	Satisfactory	Grade 1	None
3	28	Good	Good	Satisfactory	Grade 2	None
4	62	Excellent	Excellent	Anatomical	Grade 0	None
5	45	Fair	Good	Satisfactory	Grade 2	None
6	56	Excellent	Excellent	Anatomical	Grade 0	None
7	19	Poor	Fair	Satisfactory	Grade 1	None
8	33	Excellent	Excellent	Satisfactory	Grade 0	None
9	49	Good	Good	Anatomical	Grade 0	None
10	40	Poor	Fair	Unsatisfactory	Grade 1	None
11	25	Good	Good	Satisfactory	Grade 1	None

The modified Merle d'Aubigne-Postel score is a frequently used test for the functional evaluation of acetabular fractures, which evaluates pain, mobility, and gait. The modified Harris Hip score is another test frequently used in the functional evaluation of acetabulum surgical treatment, which evaluates pain, gait, and functional activities. Another functional evaluation, Clinical Grading System, was defined by Matta, and clinical scoring was done by evaluating pain, walking, and range of motion [19]. However, Md'APs and HHS, which are more frequently used in the literature, were used for functional evaluation. Matta's Reduction Criteria were used to evaluate the quality of fracture reduction radiologically. Kellgren-Lawrence radiology criteria were used to classify the severity of osteoarthritis (OA).

Surgical technique

**Figure 4:**
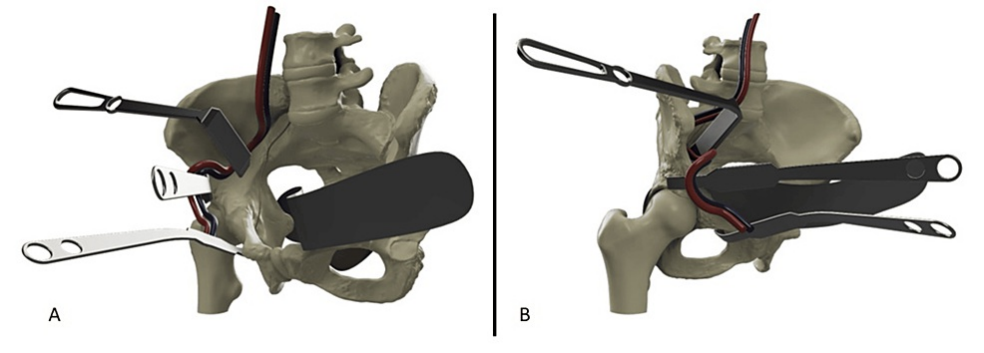
Computer-aided drawings A) iliac oblique view, B) obturator oblique view

**Figure 5:**
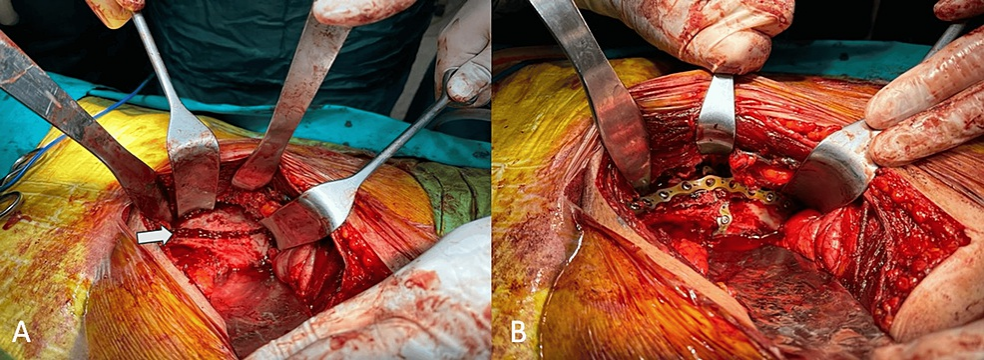
Intraoperative surgical pictures A) before osteosynthesis, B) after osteosynthesis

**Figure 6:**
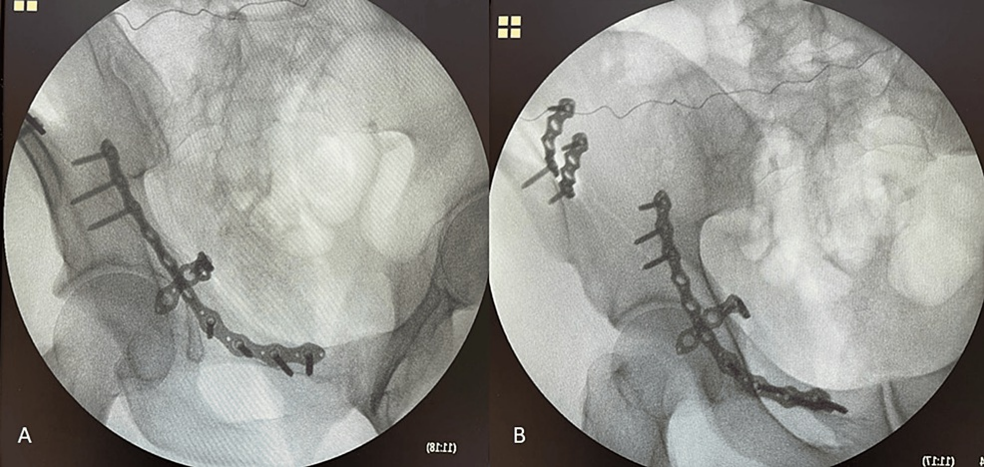
Intraoperative surgical imaging A) obturator oblique view, B) pelvis anterior posterior view

Vertical midline incision was performed from approximately 2 cm below the umbilicus to 1 cm above the symphysis pubis joint. The anterior rectus fascia was exposed and dissected. The midline splitting through the linea alba of the rectus abdominus muscle was performed. Detaching the tissues surrounding the bladder using blunt-finger dissection and mobilizing the bladder away with a malleable retractor to improve visualization, the potential space of Retzius was reached. The periosteum and iliopectineal fascia around the superior ramus and pubic root extending to the pelvic brim and the internal iliac fossa were released. During this exposure, the anastomotic branches between the internal and external iliac vessels (corona mortis) were explored and then tied or cauterized to avoid bleeding. The external iliac vessels were then located with the aid of palpation, then elevated and protected with the iliopsoas muscle by a blunt retractor without the application of excessive force [9]. After the iliopectineal fascia is incised, it is loosened both to the quadrilateral surface and to the iliopubic eminence with the help of the periosteal elevator. The area where the iliopubic eminence is located on the linea arcuata is very tough, and this area needs to be loosened a little with the help of cautery. After this loosening, scraping is done with the help of the periosteal elevator, followed by blunt dissection with the finger towards the psoas fossa, and the Blount Hohmann retractor is placed superiorly to the acetabulum, passing the psoas fossa (Figure 4). In this way, the anterior wall column is fully exposed. The important point here is that since there is a risk of thrombosis due to excessive stretching of the femoral artery-vein vascular package, attention should be paid to ensure proper circulation by loosening the Blount Hohmann retractor [9] (Figures 5, 6). If necessary, the exposure could be extended posteriorly along the pelvic brim to the sacroiliac joint. The obturator neurovascular bundle may also be located in the fatty tissue on the medial surface of the obturator internus muscle, which can then be dissected and mobilized with a retractor and another blunt-end retractor in the greater sciatic notch to achieve a better working space, especially for quadrilateral surface and posterior column fractures [9].

SPSS version 23 (IBM Inc., Armonk, New York) program was used for statistical calculations. Due to the small size of the data set and no normal distribution, the data were considered non-parametric, and the median (minimum-maximum) was used as descriptive statistics. Non-parametric data were compared using the Mann-Whitney U test; categorical data was compared using the Chi-squared test. The level of significance was considered 5% (p<0.05).

## Results

Of the 11 patients included in the study (11 out of 26 patients with acetabulum anterior wall fracture), nine were male (81.82%), and two were female (18.18%). The median age of the patients at the time of trauma was 40 (19-62), and the median time to surgery was six days (3-18), and the median operation time was 120 minutes (90-180). The median amount of intraoperative bleeding was 450 ml (200-800) according to the measurement described by the study of Açan et al. [14] (Table 1).

At the first follow-up examination at the end of the second postoperative year, the modified Merle d'Aubigné and Postel hip scores were excellent in four patients (36.37%), good in three patients (27.28%), fair in two patients (18.19%) and poor in two patients (18.19%). The modified Harris Hip score was excellent in four patients (36.37%), good in four patients (36.37%), and fair in three patients (27.28%).

According to Matta's reduction criteria, four patients (36.37%) were evaluated as anatomical, six patients (54.56%) as satisfactory, and one patient (9.1%) as unsatisfactory. According to Kellgren-Lawrence radiology criteria, five patients (45.46%) were classified as grade 0, four patients (36.37%) as grade 1, and two patients (18.19%) as grade 2 osteoarthritis.

Heterotopic ossification was not detected in any of the patients although no prophylaxis was administered. No infection was observed, and femoral nerve damage was also not detected (Table 2).

## Discussion

The most commonly used approach in anterior column and anterior wall fractures is the ilioinguinal approach [21]. The surgical technique that we used in our study describes the modified Stoppa approach in anterior acetabulum injuries. The most prominent advantages of the modified Stoppa approach are short surgical time and a low amount of bleeding. In the 30-case series of Giannoudis et al. [22], the mean surgical time was 190 minutes (40-315), while in Kelly et al.'s [23] review of 8389 cases, the mean operative time was 202.0 ± 70.3 minutes and the mean amount of blood loss was 898.6 ± 612.7 ml. The median surgical time was 120 minutes (90-180), and the median amount of blood loss was 450 ml (200-800) in our study. Although we cannot comment on the amount of bleeding due to the differences in fracture type and bleeding measurement techniques, the amount of bleeding detected in our study was lower than the surgical results obtained with the ilioinguinal approach in the literature. The median surgical time in our study is also lower than other studies in the literature.

Isolated acetabulum anterior injuries account for approximately 10% of all acetabular fractures [10-13]. In a 400-case series of acetabular fractures by Giannoudis et al., isolated acetabular anterior injuries accounted for 7.5% of all acetabular fractures. Of these patients, 86.6% (26 patients), had isolated anterior column fractures, 6.6% (two patients) had isolated anterior wall fractures, and 6.6% (two patients) had anterior wall and anterior column fractures [22]. In the review of 8389 cases by Kelly et al., the prevalence of isolated acetabulum anterior injuries was found to be 10.8%, and it was determined that 9.1% of all acetabular fractures were isolated anterior column fractures and 1.7% were isolated anterior wall fractures [23]. In a systematic review of 7876 cases of acetabular fractures in the geriatric population by Goyal et al., the prevalence of isolated anterior injury was found to be 17.23%, and it was stated that the incidence of anterior column fractures in the elderly population was higher than in younger patients [24]. In our study, out of 26 patients (10.16%) with isolated acetabulum anterior injury, 15 had isolated anterior column fracture (5.86%), eight had anterior column and anterior wall fracture (3.13%), three had isolated anterior wall fracture (1.18%), and our fracture incidences are consistent with the studies of Giannoudis et al. and Kelly et al. [22,23]. Because of the low median age of our cases, it is not possible to compare with the geriatric population incidences of Goyal et al. [24].

Giannoudis et al. analyzed the results of the anterior column, anterior wall, and anterior wall and anterior column fractures operated with the ilioinguinal approach using Matta's clinical grading system after two years of follow-up. The authors reported that, out of 30 patients, 12 patients (40%) had excellent, 11 patients (36.7%) had good, four patients (13.3%) had fair, and three patients (6.7%) had bad outcomes [22]. In our study, out of 11 patients operated with the modified Stoppa approach and evaluated with Matta's reduction criteria, four patients (36.37%) were evaluated as anatomical, six patients (54.56%) as satisfactory, and one patient (9.1%) as unsatisfactory (p=0.548). In the clinical evaluation of Giannoudis et al. using modified Merle d'Aubigne and Postel hip scores on 26 patients; eight patients (30.8%) were evaluated as excellent, eight patients (30.8%) as good, six patients (23.1%) as fair, and four patients (15.4%) as poor. In our study, evaluation using the modified Merle d'Aubigne and Postel hip score on 11 patients showed excellent outcomes in four patients (36.37%), good in three patients (27.28%), fair in two patients (18.19%), and poor in two patients (18.19%). Our clinical and radiological results are compatible with surgeries performed with the ilioinguinal approach (p=0.923). However, the results of Giannoudis et al. included isolated acetabulum anterior column fractures, and these patients were excluded from our study. Also, the modified Harris Hip score and Kellgren-Lawrence radiology criteria results could not be discussed because there is no comparable study in the literature.

There is a strong relationship between the approach used in the surgical treatment of acetabular fractures and heterotopic ossification. Firoozabadi et al. [25] concluded that the ilioinguinal approach is at low risk, the Kocher-Langenbeck approach is at medium risk, and the extended iliofemoral approach is at high risk for heterotopic ossification. Another approach used in the surgical treatment of acetabulum anterior injuries is Smith Peterson. The described modification by Lefaivre et al. [26] provides extensive access to the anterior acetabulum and the ilium. However, the risk of non-union due to iliac wing osteotomies and the risk of heterotopic ossification due to abductor muscle manipulations were noted, and each patient was offered a single dose of radiation or oral indomethacin therapy (two weeks). Although prophylaxis was not administered, heterotopic ossification was not observed in any patients. In addition, there is no risk of non-union as there is no need for osteotomy to expose the surgical site in the modified Stoppa approach.

Acetabular anterior wall fractures are rare injuries among all orthopedic traumas, and the number of cases is limited even in first-degree trauma centers. The most important shortcoming of our study is the small number of our group.

## Conclusions

The use of the modified Stoppa approach in acetabulum anterior wall fractures has shown similar results in studies with the ilioinguinal approach in the literature in terms of operation time, amount of bleeding, complication rate, and radiological and clinical results. Due to the small number of cases, we used the literature on anterior wall fractures of the acetabulum operated with the ilioinguinal approach to evaluate the functional and radiological results. Statistical comparison of these publications and our results is not possible, and this is the most important deficiency of our study.

Our main aim here is to show that the technique we describe shows similar results to the gold standard technique. We think that it is significant that this technique, which we have just described, shows clinical and radiological similar results to the gold standard technique and that a new technique could be used. We believe that it will give better clinical and radiological results than the gold standard technique in comparison with larger, multi-center case series.
